# Supplementary material for: Venetoclax Use in Paediatric Haemato-Oncology Centres in Poland: A 2022 Survey
Source: Children (Basel). 2023 Apr 19;10(4):745. doi: 10.3390/children10040745 (PMC10137026; doi:10.3390/children10040745)
Supplement: Supplementary file 1 [file children-10-00745-s001.zip › children-2294248-supplementary.pdf]

Supplementary Table S1

A list of cancer cell lines most sensitive to *BCL2* loss in a genome-wide CRISPR loss-of-function screen. Public version of DepMap Cancer Dependency Map portal at https://depmap.org/ accessed on March 5, 2023.

No	Gene Effect (Chronos)	Cel line	Disease	Age (years)	Known driver genetics
1	-1.79	SKMM2	NHL	54	no data
2	-1.29	OCILY19	NHL	27	no data
**3**	**-1.24**	**SEM**	**BCP-ALL**	**5**	***KMT2A::AFF1***
4	-1.13	KMS27	NHL	52	no data
5	-1.07	EOL1	MPN	33	no data
**6**	**-1.02**	**HB1119**	**BCP-ALL**	**<18**	***KMT2A:ENL***
7	-1.01	KARPAS1718	NHL	no data	no data
8	-0.99	KMS18	NHL	60	no data
9	-0.90	DB	NHL	45	no data
**10**	**-0.88**	**697**	**BCP-ALL**	**12**	***TCF3::PBX***
**11**	**-0.85**	**REH**	**BCP-ALL**	**15**	***ETV6::RUNX1***
12	-0.84	JJN3	NHL	57	no data
13	-0.81	OCIAML2	AML	65	*DNMT3A* R635W
14	-0.77	MOLM14	AML	20	ITD-*FLT3*
**15**	**-0.72**	**AML193**	**AML**	**13**	**no data**
**16**	**-0.72**	**NB17**	**neuroblastoma**	**1**	**no data**
**17**	**-0.65**	**LAN2**	**neuroblastoma**	**3**	**no data**
18	-0.64	TC106	Ewing sarcoma	19	*EW::ERG*
19	-0.62	RI1	NHL	57	no data
**20**	**-0.62**	**NMB**	**neuroblastoma**	**1**	**no data**

Non-Hodkgin Lymphoma, BCP-ALL –B-cell progenitor ALL, MPN –myeloproliferative neoplasm, ITD -internal tandem duplication

Pediatric cell lines are in **bold**.
